# An analysis of morphological and genetic diversity of mango fruit flies in Pakistan

**DOI:** 10.1371/journal.pone.0304472

**Published:** 2024-07-18

**Authors:** Anbareen Gul, Syed Hamid Jalal Shah, Sabyan Faris, Javaria Qazi, Atika Qazi, Samrat Kumar Dey

**Affiliations:** 1 Faculty of Biological Sciences, Department of Biotechnology, Molecular Virology and Epidemiology Laboratory, Quaid-i-Azam University, Islamabad, Pakistan; 2 Center for Agriculture and Bioscience International (CABI), Satellite Town, Rawalpindi, Pakistan; 3 Centre for Lifelong Learning, Universiti Brunei Darussalam, Brunei Darussalam; 4 School of Science and Technology (SST), Bangladesh Open University (BOU), Gazipur, Bangladesh; Sakarya Uygulamali Bilimler Universitesi, TÜRKIYE

## Abstract

Fruit flies of genus *Bactrocera* are important insect pests of commercially cultivated mangos in Pakistan limiting its successful production in the country. Despite the economic risk, the genetic diversity and population dynamics of this pest have remained unexplored. This study aimed to morphologically identify *Bactrocera* species infesting Mango in major production areas of the country and to confirm the results with insect DNA barcode techniques. Infested mango fruits from the crop of 2022, were collected from 46 locations of 11major production districts of Punjab and Sindh provinces, and first-generation flies were obtained in the laboratory. All 10,653 first generation flies were morphologically identified as two species of *Bactrocera*; *dorsalis* and *zonata* showing geography-based relative abundance in the two provinces; Punjab and Sindh. Morphological identification was confirmed by mitochondrial cytochrome oxidase gene subunit I (mt-COI) based DNA barcoding. Genetic analysis of mtCOI gene region of 61 selected specimens by the presence of two definite clusters and reliable intraspecific distances validated the results of morphological identification. This study by morphological identification of a large number of fruit fly specimens from the fields across Pakistan validated by insect DNA barcode reports two species of *Bactrocera* infesting mango in the country.

## Introduction

Fruit flies of the genus *Bactrocera* (Diptera: Tephrtitidae) with more than 5000 species are among the most important pests of fruits and vegetables in the world [1]. In addition to the polyphagous nature of some species, several are considered highly invasive; aided by globalization of trade and poor quarantine infrastructure in the developing countries. Adults often exhibit a strong tendency for dispersal and the immature stages are readily transported to new areas via fruit movement [2]. The direct damage reported by these flies is from 30 to 80% depending on the fruit variety, season, and location [3], resulting in annual losses worth billions of dollars. The cost includes both infestation and management techniques [4].

Taxonomy that utilizes morphological identification has been a gold standard for insect identification for decades [5]. However, this conventional approach is challenged by availability of taxonomic experts and keys specific to insect species, sample handling, different stages of insect metamorphosis, change in morphological traits by host adaptability and most importantly presence of hybrid species and existence of some insects as species complex [6]. DNA barcoding for insect identification including the use of mt-COI gene regions is introduced relatively recently as a parallel approach to morphology based taxonomy. It is a short standardized sequence of mt-COI gene that easily amplified by a universal set of primers and resulting sequence is able to provide a higher sequence variation at inter and intra species level [7,8].

From Pakistan eighteen species of fruit flies are morphologically characterized from different fruits and vegetables [9–15] and there are very limited reports on genetic diversity [16,17]. Similarly not much is known about the diversity and geographical distribution of fruit flies infesting Mango crop in the country [16,17].

In recent years, Pakistan has become a popular country in the production of different varieties of mango fruits and currently is the second largest mango-producing country [11,12,18]. Fruit flies are the greatest enemies of the mango fruit in Pakistan and studies from limited samples and a few areas show the presence of *B*. *dorsalis* and *B*. *zonata* from Mangos in the country [17,19,20].

This project was designed to better understand the morphological and genetic diversity of fruit flies infesting Mangos in the fields of all major Mango growing districts across Pakistan in the summer of 2022, by collecting infested fruits from the fields, rearing of fruit flies in the lab to obtain first generation and their morphological characterization validated by genetic analysis of mt-COI DNA sequence analysis.

## Material & methods

### Collection of fruit fly-infested mango samples

A team of CAB International surveyed 46 locations of 11 major Mango cultivating districts of Pakistan in summer of year 2022 from June-July. In the Punjab province 24 locations in 5 districts; Khanewal, Multan, Muzaffargarh, Rahimyar khan and Bahawalpur were surveyed [Table 1] and in the Sindh province 22 locations of 6 districts; Mirpur Khas, Sangarh, Matiari, Umarkot, Tando Allahyar and Khairpur were surveyed [Table 2].

**Table 1:** Fruit fly infested mango variety and detailed location of each sampling area of different districts of Punjab Province.

**Khanewal District**						
**Location**	**Area**	**Farmer/ field**	**Variety**	**GPS**		
				**Latitude**	**Longitude**	**Altitude**
KH-L1-A	Kabirwala	Mian Mousa gardazi	Chaunsa/Fajri	30°23"50	71°49"43	633m
KH-L1-B	Kabirwala	Pir Bilal Shah	Dosari	30°24"08	71°49"41	635m
KH-L2-A	Batiyawala	Zubair Batiyan	Fajri/Dosari	30°25"46	71°49"53	635m
KH-L2-B	Boota Kot	Haqnawaz Puni	Dosari/Malda	30°26"28	71°49"26	636m
KH-L3-A	Moosa Chak-hyderabad	Sohail Ahmed	Sindhri	30°25"29	71°42"51	633m
KH-L3-B	Ada/Salarwain, Patti Gillawali	Ashiq Gill	Chaunsa/Dosari	30°27"10	71°40"01	630m
KH-L4-A	Peeli Kothi	Nasir Jaffar Gardazi	Desi/Chaunsa, Sindhri & Langra	30°18"59	71°42"35	631m
KH-L4-B	Pul Rango	Malik Zafar	Late chaunsa, Desi Sindhri & Anwar Ratol	30°19"12	71°43"02	633m
KH-L5-A	Shor Kot	Mian Aziz	Fajri, Desi chaunsa, Anwar Ratol	30°19"24	71°52"05	650m
KH-L5-B	3 Kassi	Pir Nadeem Shah	All varieties	30°19"15	71°52"02	634m
**Multan**						
MT-L1-A	Mozah Mubarak Bucha	Haji Kala	Dosari, Anwar Ratol, Desi, Chaunsa	30°14"25	71°27"15	622m
MT-L1-B	Mozah Mubarak Bucha	Kala Bucha	Desi, Dosari, Chaunsa, Fajri	30°14"24	71°27"35	623m
MT-L2-A	Mozah Langriyal	Malik Zubair	Desi & Chaunsa	30°12"30	71°23"45	622m
MT-L2-B	Mozah-Muhammad Pur gotta	Malik Sajjad	Fajri & Desi	30°11"34	71°23"04	622m
MT-L3-A	Purrani Bastinwell-Chaka	Col. M. Akhter	Late Chaunsa, Sindhri & Anwar Ratol	29°55"20	71°18"57	617m
MT-L3-B	Mozah Derapur	Malik Niaz Ali	White Chaunsa, Late Dosari & Desi	29°58"05	71°20"20	618m
MT-L4-A	Ghari	Malik ali	Desi/Chaunsa, Dosari & Anwar Ratol	29°54"46	71°21"07	619m
MT-L4-B	Mozah-wahi Rikkhi	Abdul mana Khan	Chaunsa, Desi Dosari	29°53"50	71°20"39	620m
MT-L5-A	Basti malook	Dera Mango farm	Various varieties	29°55"27	71°30"51	619m
MT-L5-B	Chak-5 fair	Nawaz Khan	Various varieties	29°58"41	71°30"10	620m
**Muzafargarh**						
MZ-L1-A	Mozah Sonakhi	Kabir Khan	Desi, Dosari, Late Chaunsa & Fajri	30°04"25	71°16"01	589m
MZ-L1-B	Mosah Sonakhi	Jam Abdul Sattar	White Chaunsa, Late sindhari & Desi	30°03"52	71°15"24	617m
MZ-L2-A	Mozah Deen pur	Malik Nasir Bhutta	Desi, Dosari, Sindhari & Chaunsa	30°00"50	71°10"45	617m
MZ-L2-B	Thurawala-Tali mari	Sheikh Waji	Fajri, Late Chaunsa & Desi	29°59"52	71°10"47	615m
MZ-L3-A	Mozah Garewaine	Syed Javeed Shah	Desi Chaunsa, Sindhri & Anwar Ratol	29°55"13	71°07"16	615m
MZ-L3-B	Shah Jamal Road Khangarh	Haji Shafi Muhammad	Dosari, Anwar Ratol, Desi & Chaunsa	29°55"03	71°07"50	615m
MZ-L4-A	Mozah Makhanwala	MNA Ashiq Gopangh	Desi, Sindhri, Dosari & Chaunsa	29°21"01	71°00"49	604m
MZ-L4-B	Mozah Galwan doam	Malik Imdad Ali Galoo	All varieties	29°22"56	71°57"54	604m
**Bahawalpur**						
BH-L1-A	Uch-Mughallan	Sheikh Makhdoom	Dosari, Desi & Chaunsa	30°14"46	71°02"35	601m
BH-L1-B	Mozah Noor pur	Khuwaja Ansar-Zarri Model Farm	Sindhari, Anwar Ratol, Chaunsa, Dosari & Desi	29°16"01	71°02"01	601m
BH-L2-2A	Bahawalpur Road	Shahab Farm	Various varieties	29°18"40	71°33"18	615m
BH-L2-2B	Khanga Shareef	Mian Ejaz Muhammad Awaize	Various varieties	29°18"26	71°32"33	614m
BH-L3-3A	Mozah Ramzan Jariya	Kareem Bakhsh	Desi, Dosari, Anwar Ratol & Chaunsa	29°09"26	71°18"21	623m
BH-L3-B	Mozah Wahy Qadrim	Malik Azafi	Anwar Ratol, Longpal, Desi & Dosari	29°09"14	71°17"46	607m
BH-L4-A	Chamni Gotti	Saime M-Akbar	Desi, Chaunsa, Dosari & Anwar Ratol	29°05"18	71°00"58	599m
BH-L4-B	Mozah Ahmed Nesh	Sheikh Sadiq	Anwar Ratol, Faji, Chaunsa, Desi Dosari & Longpal	29°05"49	70°56"33	597m
BH-L5-A	Karar Paka Road	Major Amin	Various varieties	29°05"08	71°40"00	620m
BH-L5-B	Karar Paka Road	Major Amin	Various varieties	29°33"16	71°40"33	616m
BH-L6-A	Pulali wasti-Lodhran by-pass	Pervaiz Qureshi	Desi, Dosari, Chaunsa & Late	29°35"02	71°37"38	614m
BH-L6-B	Lodhran Multan Road	WAheed Qureshi	Dosari, Chaunsa, Desi & Longpal	29°34"56	71°37"00	615m
**Rahim Yar Khan**						
RYK-L1-A	Mozah Mubarak Bucha	Javeed Seth Akhtherabad Farm	Desi, Sindhari, Longpal & Chaunsa	28°57"05	70°43"35	604m
RYK-L1-B	Mozah Mubarak Bucha	Muhammad Akram Nasir Islah Apashi	Various varieties	28°56"42	70°43"17	595m
RYK-L2-A	Mozah Langriyal	Wazir Ahmed	Anwar Ratol, Dosari, Desi & Chaunsa	28°52"29	70°37"34	591m
RYK-L2-B	Mozah-Muhammad Pur gotta	Haji Yousaf	Anwar Ratol, Chaunsa, Desi, Fajri & Sindhari	28°51"41	70°36"14	691m
RYK-L3-A	Purrani Bastinwala-Chaka	Chaudhary Ghafoor	Anwar Ratol, Dosari, Desi & Longpal	28°45"16	70°34"23	723m
RYK-L3-B	Mozah Derapur	Dr. Mukhtageez	Anwar Ratol, Late, Desi, Mossami & 12#	28°45"04	70°35"16	596m
RYK-L4-A	Ghari	Mir Hamza	Various varieties	28°45"48	70°28"59	594m
RYK-L4-B	Mozah-wahi Rikkhi	Mir Hamza	Various varieties	28°45"32	70°29"14	591m
RYK-L5-A	Basti malook	Seth Chaudhary Javeed	All varieties	28°39"21	70°23"27	591m
RYK-L5-B	Chak-5 fair	Seth Ch Noor Ahmed	All varieties	28°39"20	70°22"21	590m

**Table 2:** Fruit fly infested mango variety and detailed location of each sampling area of different districts of Sindh Province.

**Tando Allah Yar**						
**Location**	**Area**	**Farm/field**	**Variety**	**GPS**		
				**Latitude**	**Longitude**	**Altitude**
TA-L1-A	Dhingano Buzdar	Yousaf Zulfiqar farm	Somaro	25°24"21	68°39"33	522m
TA-L1-B	Dhingano Buzdar	Yousaf Zulfiqar farm	Somaro	25°24"18.8	68°39"23.9	522m
TA-L2-A	Masan	Haji Fakhir	Somaro	25°26"27.7	68°54"23.6	523m
TA-L2-B	Masan	Haji Fakhir	Somaro	25°26"22.6	68°54"22.1	523m
TA-L3-A	Ghulam Ali	M. Ali	Somaro	25°23"59.3	68°53"09.6	518m
TA-L3-B	Ghulam Ali	M. Imran	Somaro	25°23"50	68°53"22.3	518m
TA-L4-A	Tando Somaro	Rizwan Farm	Somaro	25°32"46.3	68°39"24.7	533m
TA-L4-B	Tando Somaro	Rizwan Farm	Somaro	25°32"37.4	68°39"25	533m
TA-L5-A	Tando Somaro	Asim Farm	Somaro	25°30"50.0	68°40"37.5	526m
TA-L5-B	Tando Somaro	Asim Farm	Somaro	25°30"58.8	68°40"41	526m
TA-L6-A	Shahpur	Junaid Hyder	Somaro	25°30"26.6	68°42"33	527m
TA-L6-B	Shahpur	Junaid Hyder	Somaro	25°30"40.4	68°42"24.2	327m
**Khairpur**						
KP-L1-A	Gambat	Ghulam Dastgan	Late Sindhri, Nawab	25°35"53.4	68°42"26.41	
KP-L1-B	Gambat	Ghulam Dastgan	Nawab	27°.37"49.9	68°42"35.2	
KP-L2-A	Mahanti village	Akbar Ali	Late Chaunsa	27°41"61.8	68°37"47.3	
KP-L2-B	Mahanti village	Yousaf Ahmed	Late Chaunsa	27°41"37.2	68°37"43.2	
KP-L3-A	Waris Sambhir	Imran Sarfaraz	Nawab	27°33"57.7	68°44"31.2	
KP-L3-B	Waris Sambhir	Sajjad Haider	Nawab	27°34"09.0	68°44"35.8	
KP-L4-A	Machyoon	Iqbal Qasim	Late Sindhri, Late Chaunsa	27°34"53.6	68°43"29.6	
KP-L4-B	Machyoon	Iqbal Qasim	Late Sindhri	27°35"01.4	68°43"27.1	
KP-L5-A	Manghanwari	Ismail Ahmed	Late Sindhri	27°35"13.8	68°37"56.4	
KP-L5-B	Manghanwari	Nadeem Amin	Late Sindhri	27°35"12.0	68°39"49.6	
**Mirpur Khas**						
MK-L1-A	Mirpur Khas Road	Ch. Asghar	Somaro	25°30"31.4	69°00"08.7	
MK-L1-B	Mirpur Khas Road	Ch. Asghar	Somaro	25°.30"22.3	69°00"17.4	
MK-L2-A	Baloch Abad	Baloch farm	Somaro	25°31"07.2	69°03"33.1	
MK-L2-B	Baloch Abad	Baloch farm	Somaro	25°31"01.6	69°03"06.9	
MK-L3-A	Goth Lal Shah	Syed Ali Shah	Somaro	25°28"24.1	69°01"47	
MK-L3-B	Goth Lal Shah	Syed Ali Shah	Somaro	25°28"22.2	69°01"54.7	
MK-L4-A	Hussain Bux mari	Abbas Ahmed	Somaro, Sindhri	25°32"17.2	68°59"39.6	
MK-L4-B	Hussain Bux mari	Abbas Ahmed	Somaro, Sindhri	25°32"19.9	68°59"24.2	
MK-L5-A	Village Haji M. Bhugio	M. Umar Bhugio	Somaro	25°27"05.2	68°57"05.4	
MK-L5-B	Village Haji M. Bhugio	M. Umar Bhugio	Somaro	25°26"59.8	68°57"07.5	
**Matiari**						
MAT-L1-A	Tando Adham	Shanker Lal Mango farm	Sindhri, Somaro	25°40"49.5	68°36"41.3	
MAT-L1-B	Tando Adam	Shanker Lal Mango farm	Sindhri Somaro	25°.40"47.9	68°36"40.7	
MAT-L2-A	Kandu	Noor Ahmed	Late Sindhri	25°45"30.6	68°30"05.2	
MAT-L2-B	Kandu	Ram Lal	Late Sindhri	25°45"35.4	68°29"27.9	
**Sangarh**						
SG-L1-A	Ismail Jaguta	Haji Jam Muhammad	Sindhri, Somaro	25°43"24.6	69°06"41.5	
SG-L1-B	Ismail Jaguta	Haji Jam Muhammad	Sindhri Somaro	25°.43"21.6	69°06"39.5	
SG-L2-A	Kandiari	Shahid Ahmed	Late Sindhri	25°45"50.8	69°04"27.2	
SG-L2-B	Kandiari	Shahid Ahmed	Late Sindhri	25°45"49.9	69°04"26.3	
**Umarkot**						
UK-L1-A	Khejrari	Haji Usman Shahni	Late Sindhri	25°13"00.9	69°41"25.8	
UK-L1-B	Khejrari	Haji Usman Shahni	Late Sindhri	25°.12"55.8	69°41"22.4	

### Lab procedures to obtain first generation of fruit flies

The infested mango samples were kept in rearing room of the biological control laboratory of CAB International, with conditions of 25 ± 2 °C, 50 ± 10% RH, and a 12:12 (L:D) photoperiod. Fruit fly metamorphosis took 12–18 days and flies were observed daily for the hatching and pupation. Upon hatching, mature larvae freely left the mango fruit for pupation into a 10-15cm deep layer of moist (5–8% water) sand [21]. After pupation, the puparia were separated from the sand medium by a sieving method. The emergence of fruit flies started 4–10 days after pupation.

### Morphological description of fruit flies

The taxonomic keys of Drew and Hancock and Zubair and colleagues were used for the morphological identification [15,22] of all 10,653 specimens of first-generation flies using stereomicroscope (Nikon SMZ1000). *B*. *dorsalis* have a clear T-shaped pattern on the abdomen, their thorax is partly black and has two yellow streaks, and their body length is 6.5mm to 7.0mm while their wing has dark margins and dark anal streak. In the case of *B*. *zonata* the T-shaped pattern is absent in their abdomen, their thorax is light brown in color with two yellows streaks, their body length is 5.5 to 6.0 mm [15] while their wing have no darks margins and anal streaks.

### DNA extraction from fruit flies

For total DNA extraction from a single fruit fly Cetyl trimethyl ammonium bromide (CTAB) method was used as already reported by [16] with slight modifications. The head region of the fly was separated from the rest of the body. The head was ground with a micro-pestle in the bottom of a 1.5 mL centrifuge tube, containing 150μL lysis buffer. The lysis buffer contained 100 mM Tris-HCl, 1.4 M NaCl, 20 mM EDTA, 2% hexadecyltrimethylammonium bromide (CTAB) (Sigma-Aldrich, Darmstadt, Germany), and 7 μL of proteinase K (Fermentas). The lysate was incubated at 55°C for 1 hour. 150μL of chloroform: isoamyl-alcohol (24:1) was added, the contents were mixed by inversion, and the emulsion was separated by centrifugation, at 14,000 rpm for 10min. The DNA was precipitated by the addition of 150μL of 100% ethanol and 30μL of sodium acetate, followed by freezing at -20°C for 2 hours. The pellet was collected by microcentrifugation at 14,000 rpm for 10 min, washed with 150 μL of 70% ethanol, centrifuged again at 14,000 rpm for 10 min then air dried, dissolved in 20 μL ddH2O, and stored at −20°C.

### Amplification and sequencing of mt-COI gene region

PCR was performed using the universal primers LCO 1490-(5’-GGTCAACAAATCATAAAGATATTGG-3’), HCO 2198-(5’-TAAACTTCAGGGTGACCAAAAAATCA-3’) for 680bp mt-COI gene barcode region [23,24]. The PCR conditions were 94°C for 10 min, followed by 35 cycles at 94°C for 30 s, 49°C for 30 s, and 72°C for 45 s, with a final extension at 72°C. Each PCR reaction was performed in a volume of 25 μl containing 2.5 μl template DNA, 0.25 μl (5 U/μl) Taq polymerase (Fermentas, USA), 2.5 μl (10 X) Taq buffer (Fermentas, USA), 1.5 μl (25 mM) MgCl2 (Fermentas, USA), 2.5 μl (2mM) dNTPs (Fermentas, USA), 0.5 μl (10 pMol) forward and reverse primer and 14.75 μl ddH2O. Amplified products were resolved electrophoretically in 1% agarose gel. The size of the ladder used was 1Kb and the gel run time was 30–35 min at 80V. The DNA bands were observed under UV light in an ultraviolet (UV) trans-illuminator. The results were compared with the ladder to identify the size of the DNA sample. The PCR products were sequenced uni-directionally using forward primer by Sanger DNA sequencing (Macrogen, South Korea).

### Sequence analysis

First all the sequences were trimmed and aligned using BLAST in NCBI (https://blast.ncbi.nlm.nih.gov/Blast.cgi) to identify sequence similarities. All the sequences were truncated to the same length to eliminate missing data. The final 61 best-quality sequences of length 620 bp of mt-COI were submitted into NCBI genbank data base (accession no: OP804475-OP804502 and OP804145-OP804177. After accession numbers were assigned to our sequences genetic analysis was performed using MEGA ver. 6.0 [24] and Sequence Demarcation Tool version 1.2 [25]. Closely related sequences were retrieved from databases in FASTA format and aligned using Muscle implemented in MEGA ver. 6.0 [24], The phylogenetic tree was constructed with default values while the bootstrap value for this tree was 1000 times replicates by maximum likelihood method [26]. *Liriomyza huidobrensis* (AF327292) was used as an outgroup. To further evaluate the degree of genetic relatedness, color coded matrix and percentage pair-wise identity was generated using the Muscle algorithm in Sequence Demarcation Tool version 1. [25].

## Results

### Morphology based taxonomy of Mango fruit flies

All 10,653 first generation of fruit flies specimens that emerged from infested Mangos from all locations and districts including 7804 from Punjab and 2849 from Sindh were morphologically identified as species of Bactrocera; *dorsalis* and *zonata*. The number of *B*. *dorsalis* from Punjab samples was 2690 and *B*. *zonata* were 5114. While the number of *B*. *dorsalis* from Sindh were 2693 and *B*. *zonata* 156 indicating dominance of *B*. *dorsalis* in Sindh and *B*. *zonata* in Punjab (Tables 3 and 4).

**Table 3:** Geographical distribution of *B*. *dorsalis* and *B*. *zonata* in different districts of Punjab.

**Bahawalpur**			
**Sample name**	**Total emergence**	**No of *B*. *dorsalis***	**No of *B*. *zonata***
BH-L1-1A	40	13	27
BH-L1-1B	58	37	21
BH-L2-2A	31	10	21
BH-L2-2B	77	4	73
BH-L3-3A	62	2	60
BH-L3-3B	97	9	88
BH-L4-4A	156	11	145
BH-L4-4B	77	7	70
BH-L5-5A	61	43	18
BH-L5-5B	59	49	10
BH-L6-6A	204	20	184
BH-L6-6B	39	2	37
**Muzaffargarh**			
**Sample name**	**Total emergence**	**No of *B*. *dorsalis***	**No of *B*. *zonata***
MZ-L1-1A	76	75	1
MZ-L1-1B	81	70	11
MZ-L2-2A	19	3	16
MZ-L2-2B	47	-	47
MZ-L3-3A	383	127	256
MZ-L3-3B	202	67	135
MZ-L4-4A	25	11	14
MZ-L4-4B	171	114	57
**Khanewal**			
**Sample name**	**Total emergence**	**No of *B*. *dorsalis***	**No of *B*. *zonata***
KH-L1-1A	872	760	112
KH-L1-1B	188	127	61
KH-L2-2A	200	159	41
KH-L2-2B	222	190	32
KH-L3-3A	90	69	21
KH-L3-3B	26	23	3
KH-L4-4A	168	16	152
KH-L4-4B	210	90	120
KH-L5-5A	126	66	60
KH-L5-5B	128	22	106
**Multan**			
**Sample name**	**Total emergence**	**No of *B*. *dorsalis***	**No of *B*. *zonata***
MT-L1-1A	58	47	11
MT-L1-1B	222	140	82
MT-L2-2A	336	22	314
MT-L2-2B	206	128	78
MT-L3-3A	123	-	123
MT-L3-3B	28	7	21
MT-L4-4A	45	12	33
MT-L4-4B	49	-	49
MT-L5-5A	133	5	128
MT-L5-5B	57	7	50
**Rahimyar Khan**			
**Sample name**	**Total emergence**	**No of *B*. *dorsalis***	**No of *B*. *zonata***
RYK-L1-1A	59	2	57
RYK-L1-1B	173	-	173
RYK-L2-2A	328	35	293
RYK-L2-2B	489	69	420
RYK-L3-3A	113	3	110
RYK-L3-3B	124	-	124
RYK-L4-4A	223	-	223
RYK-L4-4B	89	-	89
RYK-L5-5A	214	14	200
RYK-L5-5B	537	3	534

**Table 4:** Geographical distribution of *B*. *dorsalis* and *B*. *zonata* in different districts of Sindh.

**Tando-Allahyar**			
**Sample**	**Total emergence**	**No of *B*. *dorsalis***	**No of *B*. *zonata***
TA-L1-1A	6	5	1
TA-L1-1B	64	54	10
TA-L2-2A	92	90	2
TA-L2-2B	18	18	-
TA-L3-3A	44	44	-
TA-L3-3B	59	59	-
TA-L4-4A	43	43	-
TA-L4-4B	8	8	-
TA-L5-5A	12	12	-
TA-L5-5B	26	26	-
TA-L6-6A	126	124	2
TA-L6-6B	8	8	-
**Mirpur Khas**			
**Sample name**	**Total emergence**	**No of *B*. *dorsalis***	**No of *B*. *zonata***
MK-L1-1A	4	4	-
MK-L1-1B	13	13	-
MK-L2-2A	34	32	2
MK-L2-2B	106	99	7
MK-L3-3A	156	141	15
MK-L3-3B	20	18	2
MK-L4-4A	99	92	7
MK-L4-4B	32	27	5
MK-L5-5A	36	36	-
MK-L5-5B	19	19	-
**Matiari**			
**Sample name**	**Total emergence**	**No of *B*. *dorsalis***	**No of *B*. *zonata***
MAT-L1-1A	55	50	5
MAT-L1-1B	64	58	6
MAT-L2-2A	45	41	4
MAT-L2-2B	34	18	16
**Khairpur**			
**Sample name**	**Total emergence**	**No of *B*. *dorsalis***	**No of *B*. *zonata***
KP-L1-1A	223	223	-
KP-L1-1B	165	161	4
KP-L2-2A	-	-	-
KP-L2-2B	212	206	6
KP-L3-3A	345	334	11
KP-L3-3B	2	2	-
KP-L4-4A	117	117	-
KP-L4-4B	106	102	4
KP-L5-5A	13	13	-
KP-L5-5B	132	130	2
**Sangarh**			
**Sample name**	**Total emergence**	**No of *B*. *dorsalis***	**No of *B*. *zonata***
SG-L1-1A	23	23	-
SG-L1-1B	6	-	6
SG-L2-2A	39	6	33
SG-L2-2B	51	45	6
**Umarkot**			
**Sample name**	**Total emergence**	**No of *B*. *dorsalis***	**No of *B*. *zonata***
UK-L1-1A	153	153	-
UK-L1-1B	39	39	-

### Vallidation of morphological identification by analysis of mtCOI sequence diversity

The amplification of the mt-COI region of fruit flies was achieved from randomly selected single fruit flies from each sample and sequenced. BLAST analysis of 61 representative sequences showed its homology with mt-COI sequences of two respective species *Bactrocera*; *B*. *dorsalis* (India MN016995 and China MG689732 isolates) and *B*. *zonata* (Iran MG881714, MG881760 and China MG962410 isolates) confirming the morphological identification. Sequence data of two reference samples of *B*. *dorsalis* and *B*. *zonata* taken from the CAB International library were also included in analysis as control.

Inter and intraspecific genetic diversity among *B*. *dorsalis* and *B*. *zonata* specimens was also analysed using MEGA6. The intraspecific diversity among *B*. *dorsalis* was 0.011% and *B*. *zonata* was 0.016% indicating fewer variations in intraspecific diversity while the interspecific genetic diversity among these two species collected from Punjab and Sindh province were 0.117%. The software calculated interspecific and intraspecific genetic diversity by measuring the genetic differences (e.g., nucleotide substitutions) among these sequences and by calculating parameters like nucleotide diversity (π), which quantifies the average number of nucleotide differences between two sequences.

**Fig 1:**
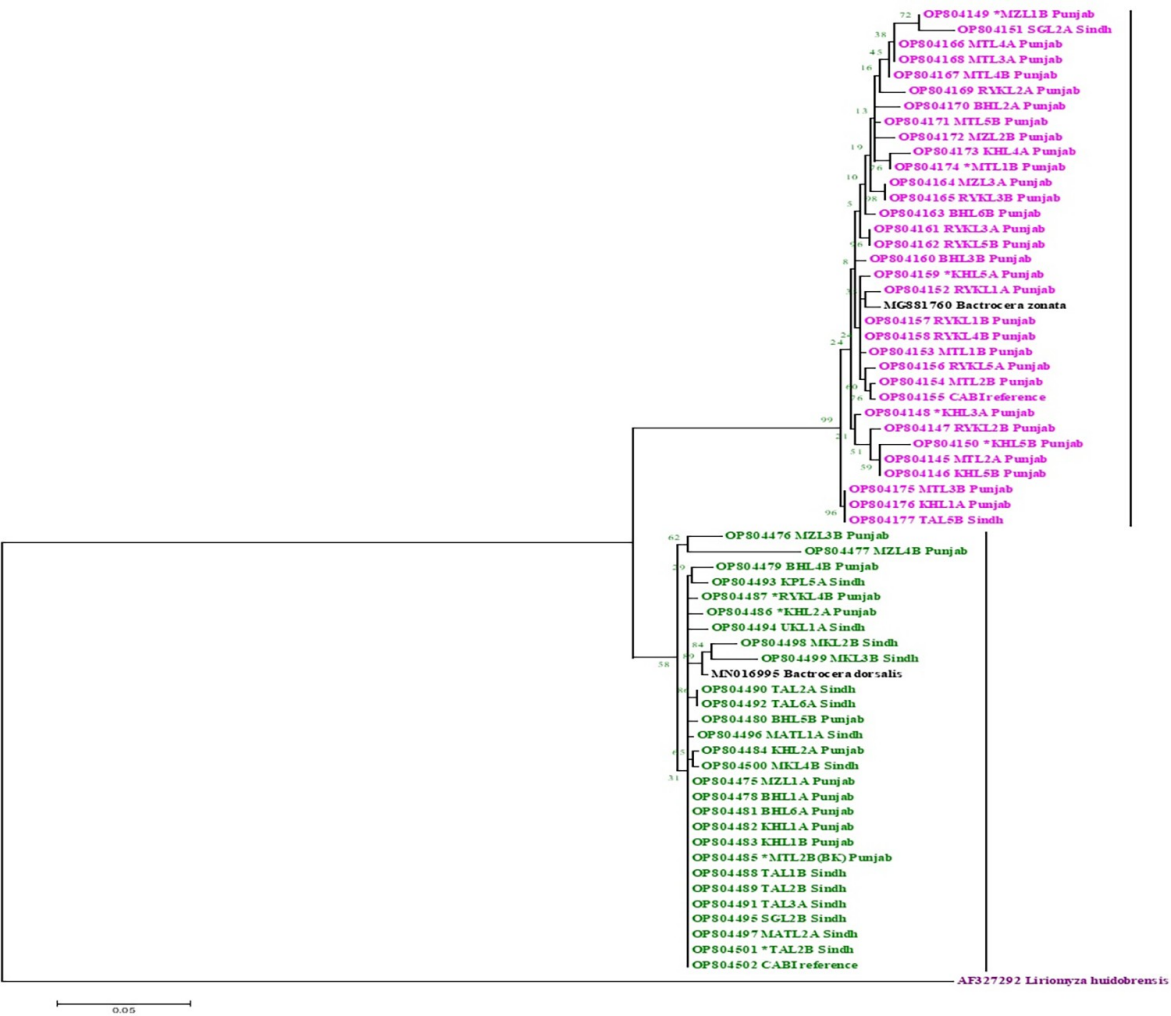
Phylogenetic tree showing the relationship among fruit flies based on mitochondrion cytochrome oxidase I (mt-COI) gene. The sequence Phylogenetic tree showing the relationship among fruit flies based on mitochondrion cytochrome oxidase I (mt-COI) gene. The sequence of AF327292 *Liriomyza huidobrensis* specie was used as out group. This tree was constructed using maximum likelihood tree on Mega 6.

For the phylogenetic tree construction, no insertions, deletions, or stop codons were present in the alignment. These sequences were aligned using n-BLAST then sequences with accession no: OP804475-OP804502 showed 96–100% similarity with already stored sequences in the database while the sequences with accession no: OP804145-OP804177 showed 98–100% similarity. One closely related sequence of COI genes of both *B*. *dorsalis* and *B*. *zonata* were downloaded from databases in FASTA format and used in MEGA 6 software to analyze phylogenetic analysis by maximum likelihood method. Phylogenetic analysis in Fig 1 shows our sequences clustered in two groups: *B*. *dorsalis* and *B*. *zonata*. Where *Liriomyza huidobrensis* (AF327292) was used as an outgroup.

## Matrix analysis of sequences

**Fig 2:**
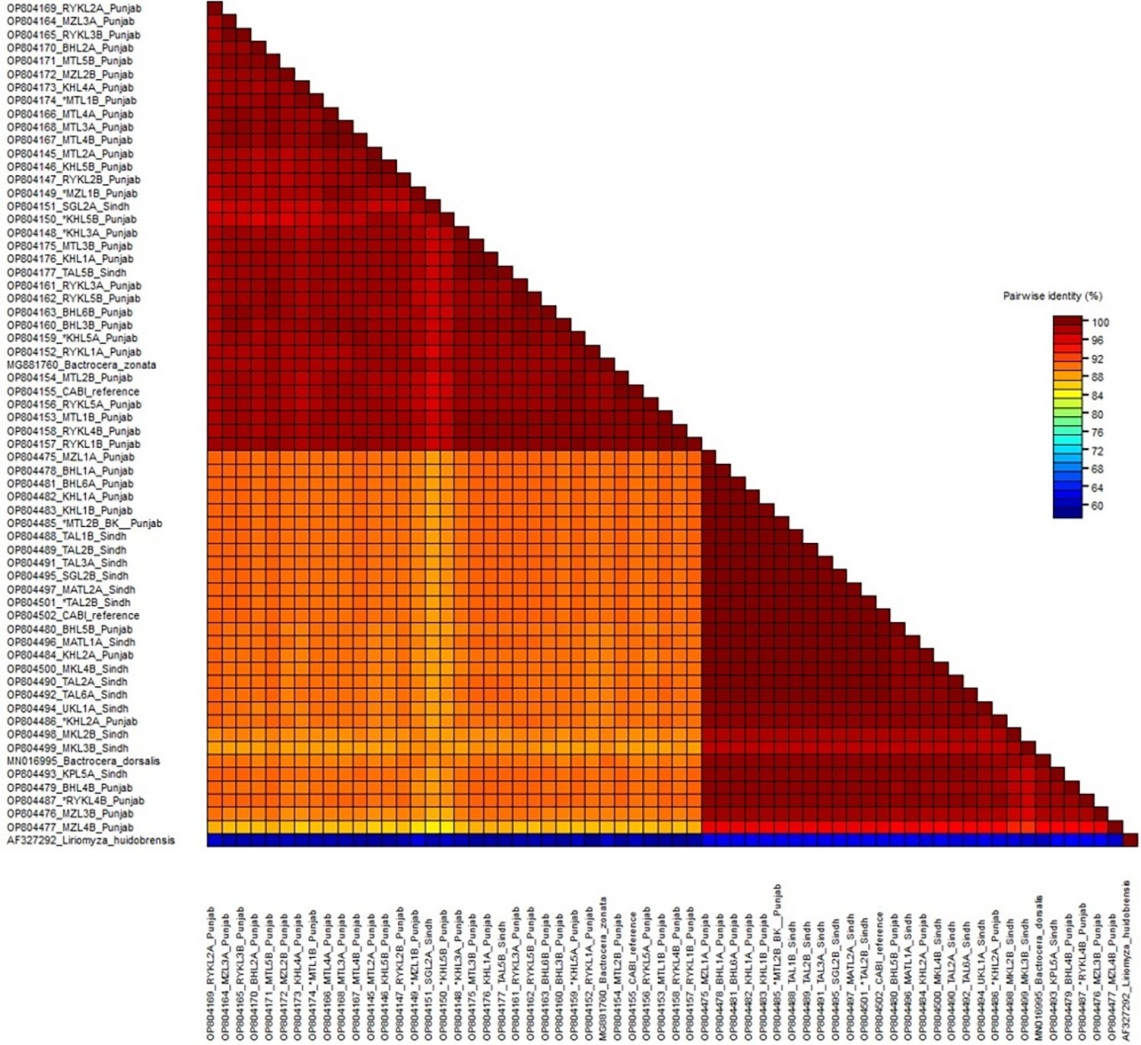
This color-coded pairwise identity matrix was generated from 61 sequences of fruit fly samples from different districts of Punjab and Sindh. Each colored cell represents a percentage identity score between two sequences (one horizontally to the left and the other vertically at the bottom). A colored key displayed in the matrix indicates the percentage pairwise identity.

For the validation of phylogenetic analysis, matrix analysis of the sequences was also performed using the sequence demarcation tool (SDTv1.2) as shown in Fig 2 representing two clusters. The pair-wise sequence identity score of all fruit fly sequences from Punjab province showed 100–89% similarity among each other while their similarity score with MG881760 *B*. *zonata* reported from Iran is 97–98% Table 5. Table 6 indicates the fruit fly sequences showing the pair-wise sequence identity score of 100–96% among each other and with MN016995 *B*. *dorsalis* sequence reported from India is 100–90%.

**Table 5:** Pairwise sequence identity analysis of mt-COI gene of *B*. *zonata* sequences using sequence demarcation tool version 1.2 (SDTv1.2).

OP804169_RYKL2A_Punjab	100																																	
OP804164_MZL3A_Punjab	98	100																																
OP804165_RYKL3B_Punjab	98	100	100																															
OP804170_BHL2A_Punjab	98	99	99	100																														
OP804171_MTL5B_Punjab	99	99	99	99	100																													
OP804172_MZL2B_Punjab	98	99	99	98.5	99.2	100																												
OP804173_KHL4A_Punjab	98	99	99	98.1	98.7	98.5	100																											
OP804174_*MTL1B_Punjab	98	99	99	98.7	99.4	98.9	99.4	100																										
OP804166_MTL4A_Punjab	99	99	99	98.5	99.2	98.7	98.5	98.9	100																									
OP804168_MTL3A_Punjab	99	99	99	98.5	99.2	98.7	98.5	98.9	100	100																								
OP804167_MTL4B_Punjab	99	99	99	98.7	99.4	98.9	98.7	99	99.8	100	100																							
OP804145_MTL2A_Punjab	98	98	98	97.4	98.1	97.6	97.7	97.7	98.5	99	98.7	100																						
OP804146_KHL5B_Punjab	98	98	98	97.4	98.1	97.6	97.7	97.7	98.5	99	98.7	100	100																					
OP804147_RYKL2B_Punjab	97	98	98	97.4	98.1	97.6	97.7	97.7	98.5	99	98.7	99.4	99.4	100																				
OP804149_*MZL1B_Punjab	98	98	98	97.6	98.2	97.7	97.9	97.9	99	99	98.9	97.9	97.9	97.9	100																			
OP804151_SGL2A_Sindh	96	97	97	96.4	97.1	96.6	96.8	96.8	97.7	98	97.6	96.6	96.6	96.6	98.7	100																		
OP804150_*KHL5B_Punjab	96	97	97	96.1	96.8	96.3	96.4	96.4	97.3	97	97.4	98.7	98.7	98.1	97.6	97.9	100																	
OP804148_*KHL3A_Punjab	98	98	98	97.9	98.5	98.1	97.6	98.2	98.7	99	98.9	98.9	98.9	98.9	97.7	96.4	97.6	100																
OP804175_MTL3B_Punjab	98	98	98	98.4	98.9	98.4	97.9	98.5	98.7	99	98.9	98.5	98.5	98.5	97.7	96.4	97.3	99	100															
OP804176_KHL1A_Punjab	98	98	98	98.4	98.9	98.4	97.9	98.5	98.7	99	98.9	98.5	98.5	98.5	97.7	96.4	97.3	99	100	100														
OP804177_TAL5B_Sindh	98	98	98	98.4	98.9	98.4	97.9	98.5	98.7	99	98.9	98.5	98.5	98.5	97.7	96.4	97.3	99	100	100	100													
OP804161_RYKL3A_Punjab	98	99	99	98.4	99	98.5	98.1	98.7	98.5	99	98.7	98.4	98.4	98.7	97.6	96.4	97.1	98.9	98.9	98.9	98.9	100												
OP804162_RYKL5B_Punjab	98	99	99	98.4	99	98.5	98.1	98.7	98.5	99	98.7	98.4	98.4	98.7	97.6	96.4	97.1	98.9	98.9	98.9	98.9	100	100											
OP804163_BHL6B_Punjab	98	99	99	98.5	99.2	98.7	98.2	98.9	98.7	99	98.9	98.2	98.2	98.2	97.7	96.6	96.9	98.7	98.7	98.7	98.7	99.2	99.2	100										
OP804160_BHL3B_Punjab	98	99	99	98.2	98.9	98.4	98.5	98.9	99	99	99.2	98.9	98.9	98.9	98.1	96.8	97.6	99	99	99	99	99.2	99.2	99	100									
OP804159_*KHL5A_Punjab	98	99	99	98.1	98.7	98.2	98.1	98.4	98.9	99	99	98.7	98.7	98.7	97.9	96.6	97.4	98.9	98.9	98.9	98.9	99	99	98.9	99.5	100								
OP804152_RYKL1A_Punjab	98	98	98	97.7	98.4	97.9	97.7	98.1	98.2	98	98.4	98.7	98.7	98.7	97.6	96.3	97.4	98.5	98.5	98.5	98.5	98.7	98.7	98.5	98.9	99	100							
MG881760_Bactrocera_zonata	98	98	98	97.7	98.4	97.9	97.7	98.1	98.2	98	98.4	98.4	98.4	98.4	98.5	97.3	98.1	98.5	98.5	98.5	98.5	98.7	98.7	98.5	98.9	99	99	100						
OP804154_MTL2B_Punjab	98	98	98	97.9	98.5	98.1	97.6	98.2	98.4	98	98.5	98.2	98.2	98.2	97.4	96.1	96.9	98.7	98.7	98.7	98.7	98.9	98.9	98.7	99	99.2	98.9	98.9	100					
OP804155_CABI_reference	98	98	98	97.9	98.5	98.1	97.6	98.2	98.4	98	98.5	98.2	98.2	98.2	97.4	96.1	96.9	98.7	98.7	98.7	98.7	98.9	98.9	98.7	99	99.2	98.9	98.9	99.7	100				
OP804156_RYKL5A_Punjab	98	98	98	97.9	98.5	98.1	97.6	98.2	98.4	98	98.5	98.5	98.5	98.2	97.4	96.1	97.3	98.7	98.7	98.7	98.7	98.9	98.9	98.7	99	99.2	98.9	98.9	99.4	99.4	100			
OP804153_MTL1B_Punjab	98	99	99	98.2	98.9	98.4	97.9	98.5	98.7	99	98.9	98.5	98.5	98.5	97.7	96.4	97.2	99	99	99	99	99.2	99.2	99	99.4	99.5	99.2	99.2	99.4	99.4	99.4	100		
OP804158_RYKL4B_Punjab	98	99	99	98.2	98.9	98.4	97.9	98.5	98.7	99	98.9	98.5	98.5	98.5	97.7	96.4	97.3	99	99	99	99	99.2	99.2	99	99.4	99.5	99.2	99.2	99.4	99.4	99.4	99.7	100	
OP804157_RYKL1B_Punjab	98	99	99	98.4	99	98.5	98.1	98.7	98.9	99	99	98.7	98.7	98.7	97.9	96.6	97.4	99.2	99.2	99.2	99.2	99.4	99.4	99.2	99.5	99.7	99.4	99.4	99.5	99.5	99.5	99.8	99.8	100

**Table 6:** Pairwise sequence identity analysis of mt-COI gene of *B*. *dorsalis* sequences using sequence demarcation tool version 1.2 (SDTv1.2).

OP804475_MZL1A_Punjab	100																													
OP804478_BHL1A_Punjab	100	100																												
OP804481_BHL6A_Punjab	100	100	100																											
OP804482_KHL1A_Punjab	100	100	100	100																										
OP804483_KHL1B_Punjab	100	100	100	100	100																									
OP804485_*MTL2B_BK__Punjab	100	100	100	100	100	100																								
OP804488_TAL1B_Sindh	100	100	100	100	100	100	100																							
OP804489_TAL2B_Sindh	100	100	100	100	100	100	100	100																						
OP804491_TAL3A_Sindh	100	100	100	100	100	100	100	100	100																					
OP804495_SGL2B_Sindh	100	100	100	100	100	100	100	100	100	100																				
OP804497_MATL2A_Sindh	100	100	100	100	100	100	100	100	100	100	100																			
OP804501_*TAL2B_Sindh	100	100	100	100	100	100	100	100	100	100	100	100																		
OP804502_CABI_reference	100	100	100	100	100	100	100	100	100	100	100	100	100																	
OP804480_BHL5B_Punjab	99.7	99.7	99.7	99.7	99.7	99.7	99.7	99.7	99.7	99.7	99.7	99.7	99.7	100																
OP804496_MATL1A_Sindh	99.8	99.8	99.8	99.8	99.8	99.8	99.8	99.8	99.8	99.8	99.8	99.8	99.8	99.5	100															
OP804484_KHL2A_Punjab	99.7	99.7	99.7	99.7	99.7	99.7	99.7	99.7	99.7	99.7	99.7	99.7	99.7	99.4	99.5	100														
OP804500_MKL4B_Sindh	99.7	99.7	99.7	99.7	99.7	99.7	99.7	99.7	99.7	99.7	99.7	99.7	99.7	99.4	99.5	99.7	100													
OP804490_TAL2A_Sindh	99.7	99.7	99.7	99.7	99.7	99.7	99.7	99.7	99.7	99.7	99.7	99.7	99.7	99.4	99.5	99.4	99.4	100												
OP804492_TAL6A_Sindh	99.7	99.7	99.7	99.7	99.7	99.7	99.7	99.7	99.7	99.7	99.7	99.7	99.7	99.4	99.5	99.4	99.4	100	100											
OP804494_UKL1A_Sindh	99.4	99.4	99.4	99.4	99.4	99.4	99.4	99.4	99.4	99.4	99.4	99.4	99.4	99	99.2	99	99	99.2	99.2	100										
OP804486_*KHL2A_Punjab	99.5	99.5	99.5	99.5	99.5	99.5	99.5	99.5	99.5	99.5	99.5	99.5	99.5	99.2	99.4	99.2	99.2	99.2	99.2	98.9	100									
OP804498_MKL2B_Sindh	98.2	98.2	98.2	98.2	98.2	98.2	98.2	98.2	98.2	98.2	98.2	98.2	98.2	97.9	98.1	97.9	97.9	98.1	98.1	97.6	97.7	100								
OP804499_MKL3B_Sindh	97.4	97.4	97.4	97.4	97.4	97.4	97.4	97.4	97.4	97.4	97.4	97.4	97.4	97.1	97.3	97.1	97.1	97.1	97.1	97.1	97.1	97.8	100							
MN016995_Bactrocera_dorsalis	99.4	99.4	99.4	99.4	99.4	99.4	99.4	99.4	99.4	99.4	99.4	99.4	99.4	99	99.2	99	99	99	99	98.7	98.9	98.4	97.9	100						
OP804493_KPL5A_Sindh	99.4	99.4	99.4	99.4	99.4	99.4	99.4	99.4	99.4	99.4	99.4	99.4	99.4	99	99.2	99	99	99	99	98.7	98.9	97.6	96.8	98.7	100					
OP804479_BHL4B_Punjab	99.2	99.2	99.2	99.2	99.2	99.2	99.2	99.2	99.2	99.2	99.2	99.2	99.2	98.9	99	98.9	98.9	98.9	98.9	98.5	98.7	97.4	96.6	98.5	98.9	100				
OP804487_*RYKL4B_Punjab	99.5	99.5	99.5	99.5	99.5	99.5	99.5	99.5	99.5	99.5	99.5	99.5	99.5	99.2	99.4	99.2	99.2	99.2	99.2	98.9	99	97.7	96.9	98.9	98.9	98.7	100			
OP804476_MZL3B_Punjab	98.4	98.4	98.4	98.4	98.4	98.4	98.4	98.4	98.4	98.4	98.4	98.4	98.4	98.1	98.5	98.1	98.1	98.1	98.1	97.7	97.9	96.6	95.8	97.7	97.7	97.9	97.9	100		
OP804477_MZL4B_Punjab	95.3	95.3	95.3	95.3	95.3	95.3	95.3	95.3	95.3	95.3	95.3	95.3	95.3	95	95.5	95	95	95	95	94.7	94.8	93.5	92.7	94.7	94.7	94.5	94.8	95.3	100	
AF327292_Liriomyza_huidobrensis	62.1	62.1	62.1	62.1	62.1	62.1	62.1	62.1	62.1	62.1	62.1	62.1	62.1	62.1	61.9	61.7	62.1	61.5	61.5	62.3	61.5	61.4	61	60.3	62.4	62.8	62.9	62.7	60.9	100

## Discussion

Morphological identification of insect pests is a classical and basic method of insect identification. This method has some limitations i.e. most of the economically important pests are difficult to identify via morphometric keys even by specialists as a large number of insect pests belong to morphologically cryptic species [27]. Also, identifications of eggs and instars of pest species are difficult to determine morphologically [28]. Compared to this, DNA barcoding provides a quick and authentic means for species identification [29]. However, understanding the population structure and genetic diversity of insect-like fruit flies in any region requires the characterization of a large number of insects. The process of DNA barcode is expensive and the cost is a major challenge in determining the large population structures of these insects [29]. Therefore, in this study, we collected infested mango samples across mango growing areas of Pakistan and first morphologically characterized all first-generation fruit flies. Then we randomly selected fruit flies from each location for mt-COI-based DNA barcode analysis and verified our morphometric data. This approach allowed us to provide barcode validation of morphologically characterized 10,653 fruit fly samples infesting commercially cultivated mangos in Pakistan.

Dominance of *B*. *zonata* in Punjab is reported earlier from the country by small-scale studies from limited locations and samples [19]. There are only three reports on very limited genetic diversity [9,16,17]. Similarly, no report has been published before on diversity and geographical distribution of fruit flies infesting Mango crop in the country. Both these species are also reported as major pests of mango from Bangladesh and neighboring countries of Pakistan; Iran and India [30–33].

To ascertain genetic diversity, we used MEGA ver. 6 that calculated interspecific and intraspecific genetic diversity by measuring the genetic differences (e.g., nucleotide substitutions) among these sequences and by calculating parameters like nucleotide diversity (π), which quantifies the average number of nucleotide differences between two sequences. The methodology involved quantifying genetic variation within and between species. Intraspecific genetic diversity to be 0.011% for *B*. *dorsalis* and 0.016% for *B*. *zonata*. These values point to a relatively limited degree of genetic variability within their respective conspecific populations. In contrast, the interspecific genetic diversity between these two species, as sampled from the Punjab and Sindh provinces, demonstrated a notably higher value, measuring 0.117%. This observation underscores the more pronounced genetic disparities that exist between these distinct species.

In a comparable fashion, when the genetic sequences of *B*. *zonata* and *B*. *dorsalis* collected from both the province were subjected to a BLAST analysis, and when they were compared with sequences from neighboring countries, such as Iran and India. The results of this analysis revealed a striking degree of genetic similarity, with sequence identities of 97–98% for MG881760 and 100–90% for MN016995 respectively, thus highlighting the presence of discernible genetic variations among the fruit fly populations of Pakistan and its adjacent countries.

This study is the first of its kind in Pakistan endeavoring survey of the geographical locations across two major mango producing province of the country with the specific objectives of fruit fly identification and genetic analysis. The outcomes of this research offer a significant and novel contribution by furnishing crucial insights into the genetic diversity of this economically significant pest species to help in its pest management.

## Conclusion

The morphology-based identification validated by mt-COI gene barcoding shows the presence of two species of *Bactrocera* in mango crop of year 2022 in Pakistan. Validation of morphological data by mt-COI gene analysis shows that in the absence of barcode technology morphology-based identification is an effective approach for screening adult fruit flies of *Bactrocera* genus. However, integrated barcoding, a combination of traditional taxonomy and molecular methods, enhances the accuracy and reliability of results. Overall, this study contributes important information on species diversity and genetic variation of *Bactrocera* on Mango crop in Pakistan.
